# Genomic and Transcriptomic Survey Provides New Insight into the Organization and Transposition Activity of Highly Expressed LTR Retrotransposons of Sunflower (*Helianthus annuus* L.)

**DOI:** 10.3390/ijms21239331

**Published:** 2020-12-07

**Authors:** Ilya Kirov, Murad Omarov, Pavel Merkulov, Maxim Dudnikov, Sofya Gvaramiya, Elizaveta Kolganova, Roman Komakhin, Gennady Karlov, Alexander Soloviev

**Affiliations:** 1 All-Russia Research Institute of Agricultural Biotechnology, Timiryazevskaya Str. 42, 127550 Moscow, Russia; muradok98@gmail.com (M.O.); paulmerkulov97@gmail.com (P.M.); max.dudnikov.07@gmail.com (M.D.); sofia.gvaramia@gmail.com (S.G.); liza.colg@gmail.com (E.K.); komakhin@gmail.com (R.K.); karlovg@gmail.com (G.K.); A.Soloviev70@gmail.com (A.S.); 2 Kurchatov Genomics Center of ARRIAB, All-Russia Research Institute of Agricultural Biotechnology, Timiryazevskaya Street, 42, 127550 Moscow, Russia; 3 Faculty of Computer Science, National Research University Higher School of Economics, Pokrovsky Boulvar 11, 109028 Moscow, Russia

**Keywords:** retrotransposons, nanopore, transcription, mobilome, sunflower, gag, splicing

## Abstract

LTR retrotransposons (RTEs) play a crucial role in plant genome evolution and adaptation. Although RTEs are generally silenced in somatic plant tissues under non-stressed conditions, some expressed RTEs (exRTEs) escape genome defense mechanisms. As our understanding of exRTE organization in plants is rudimentary, we systematically surveyed the genomic and transcriptomic organization and mobilome (transposition) activity of sunflower (*Helianthus annuus* L.) exRTEs. We identified 44 transcribed RTEs in the sunflower genome and demonstrated their distinct genomic features: more recent insertion time, longer open reading frame (ORF) length, and smaller distance to neighboring genes. We showed that GAG-encoding ORFs are present at significantly higher frequencies in exRTEs, compared with non-expressed RTEs. Most exRTEs exhibit variation in copy number among sunflower cultivars and one exRTE *Gagarin* produces extrachromosomal circular DNA in seedling, demonstrating recent and ongoing transposition activity. Nanopore direct RNA sequencing of full-length RTE RNA revealed complex patterns of alternative splicing in RTE RNAs, resulting in isoforms that carry ORFs for distinct RTE proteins. Together, our study demonstrates that tens of expressed sunflower RTEs with specific genomic organization shape the hidden layer of the transcriptome, pointing to the evolution of specific strategies that circumvent existing genome defense mechanisms.

## 1. Introduction

LTR retrotransposons (RTEs) are mobile genetic elements capable of transposition via RNA intermediates. RTE RNAs encode proteins required for virus-like particle formation, cDNA synthesis, and integration. They share similarities with retroviruses with respect to their structural features (i.e., two identical long-terminal repeats (LTRs) and one open reading frame that encodes all the proteins produced by the RTE), life cycle, and origin of the encoded proteins.

RTEs play a key role in animal and plant genome evolution by modulating gene transcription, genome architecture and providing “raw material” for evolutionary novelties [1,2,3]. RTE transcription and transposition are epigenetically silenced due to the potential negative impact they can have on short-term plant fitness [4,5,6,7]. Because the genome protects itself from RTEs using a multipronged strategy, transcription and transposition of RTEs in non-stressed conditions are widely thought to be the exception rather than the rule. However, recent genome-wide surveys of RTE expression in animals and plants indicate that thousands of retrotransposons have detectable expressions [8,9]. This led to the coining of the term “retrotranscriptome” to describe the complement of RTE transcripts in a cell [10]. Transcripts derived from transposable elements (TEs) may account for a substantial number of RNA-seq reads and EST (Expressed Sequence Tags) sequences in plants and animals [8,11,12,13,14,15]. TE-derived transcripts comprised up to 23.44% of all mapped RNAseq reads in the opossum spleen. Genome-wide RTE expression analysis based on RNA-seq data established that up to 10.38% and 26.1% of all mapped reads were associated with long-terminal repeat (LTR) retrotransposons in tea tree (*Camellia sinensis*) and maize (*Zea mays* L.), respectively [15]. In plants, some tissues, such as the endosperm, have reduced levels of DNA methylation that may trigger transposon activity [16,17,18,19]. Several lines of evidence suggest that RTE expression is elevated in reproductive tissues—a phenomenon common to monocots and dicots [8,12,20]. A survey of maize TE expression found that 4.4% of TEs were differentially expressed during male gametophyte development [12]. Thus, the environment of developing pollen cells favors TE expression.

The stochastic model of genome transcription may explain the low genome-wide transcription of RTEs in some cases [21]. However, for several reasons, it does not explain the majority of cases. First, RTE transcription and genome abundance do not correlate well with each other [11]. Second, transcription of RTEs can be stage- and tissue-specific; the number of detectable RTEs varies across developmental stages, organs, and stress conditions [14,17,18,20]. An increasing number of RTEs, which are repressed under non-stress conditions, become transcriptionally and transpositionally active in stress [22,23].

Understanding the genomic features and biases specific for transcribed RTEs versus non-transcribed ones may reveal how RTE expression occurs despite the activity of silencing mechanisms. Genome-wide characterization studies in *Arabidopsis thaliana* and maize show that expressed RTEs are generally longer than those that are not expressed [7,12,22]. In addition, heat-activated TEs in *A. thaliana* have a higher GC content and higher levels of the heterochromatic histone mark H3K9me2, than non-expressed TEs [22]. Expressed TEs in maize are distributed throughout the genome, near actively expressing genes, in some tissues [12]. However, it is not clear if TE expression influences neighboring gene expression or vice versa [12].

The transcription level of RTEs is not directly linked to their transposition activity [16]. RTE transposition is a multistep procedure that requires a full set of RTE proteins including GAG, Reverse transcriptase, RNAse H, Integrase and aspartyl proteases as well as intact RTE genomic RNA (gRNA). Moreover, the number of GAG protein molecules must exceed the number of other RTE proteins by more than an order of magnitude. RTEs of the same family can dramatically differ in transposition activity, while having similar levels of transcriptional activity. For example, members of the Osr4 family in rice are transcriptionally active in the endosperm, but only the members of the PopRice subfamily form extrachromosomal circular DNA (eccDNA), a signature of transposition activity [16]. The mechanisms that limit the mobility of transcriptionally active retrotransposons are poorly understood.

RTEs form their own ecosystem in a cell; the various groups differ in their expression patterns. One group comprises TEs that become active in certain stress conditions and developmental stages, when the general rate of genome defense against RTEs is lower. Another group consists of TEs that are deactivated only when the genes involved in genome defense (such as *DDM1*, *RDR6,* etc.) are mutated. The third group includes TEs with ubiquitous expression in vegetative tissues and non-stress conditions. The latter group is the most purely studied up to date and the number of such RTEs characterized in plants has only recently been increased thanks to new algorithms and sequencing technologies. We do not yet know which features distinguish these RTEs from others with regard to genomic and transcriptomic levels.

RTE transcription is often ignored during genome annotation because RTE-derived RNA-seq reads may generate multiple hits during genome mapping. In addition, read-through transcription from neighboring genes, alternative transcription start- and stop-sites, pieces of RTEs embedded into transcribed genes, and solo-LTRs may all result in RTE-related RNA-seq reads that do not reflect RTE transcription status. For example, the bulk of RTE-related transcripts in tea are not similar to known RTE domains [15], suggesting that these transcripts may only have short RTE-related sequences, rather than being transcripts of the full-length RTEs. Although some algorithms (reviewed by [24]) address this issue, the noisy RNAseq RTE-related reads complicate RTE transcription identification. No algorithms exist that reconstruct entire TE transcripts. Nanopore RNA (cDNA) sequencing has recently been applied to plant TE “gene-like” annotation in an *Arabidopsis* mutant defective in the main TE-silencing pathways [25]. The nanopore cDNA reads from *Arabidopsis* and the PacBio reads from maize can be exploited to precisely determine TE transcripts and transcription features, including transcription start sites and poly-A tail length. This pioneering study demonstrated the opportunities offered by nanopore sequencing to decipher the structure and processing of RTE RNAs.

In this study, we aimed to depict the distinct genomic and transcriptomic features and to obtain insight into the mobilome (transposition) activity of expressed RTEs. For this, we exploited sunflower (*Helianthus annuus*), a species with a big genome (~3 Gb), and for which RTE transcription has been shown in several previous studies [26,27,28,29,30,31]. Previous reports on RTE transcription in sunflower focused on distinct TE elements isolated from BAC clones, or those assembled by short-read clustering; they did not include genome-based characterization of expressed RTEs (exRTEs) and their transcripts. Our genome-wide analysis allowed us to identify 44 exRTEs with significant levels of expression in stressed and/or non-stressed conditions. We show that exRTEs are distinguished from their non-expressed counterparts by distinct genomic features, such as a recent insertion time, proximity to genes, low copy number, and enrichment in open reading frames (ORFs) encoding a GAG domain with one RNA-binding domain. Using bioinformatic screening of whole-genome data from 13 cultivars, we determined the transposition activity for 63% of exRTEs. Moreover, we analyzed eccDNA and found, for the first time in this species, one exRTE called *Gagarin* that demonstrates ongoing mobilome activity. Moreover, we used nanopore direct RNA sequencing in seedlings of two cultivars, to show that some exRTEs undergo splicing, which results in a complex mixture of isoforms encoding different sets of proteins. Altogether, our study uncovers a substantial contribution of RTEs to transcriptome complexity and reveals a unique set of expressed and active retrotransposons that could aid future studies.

## 2. Results

### 2.1. Tens of Sunflower LTR Retrotransposons Have High Expression in Stress and Normal Conditions

**Figure 1:**
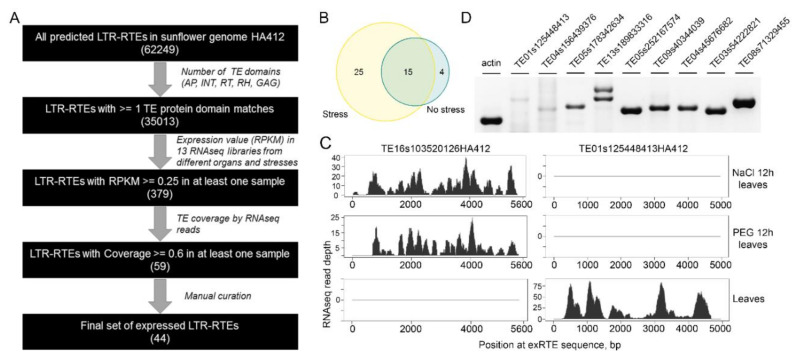
RNAseq-based identification of expressed LTR retrotransposons (exRTEs) in sunflower. (**A**): Scheme of the pipeline used in this study. (**B**): Venn diagram showing the number of exRTEs with detectable expression in stressed and non-stressed conditions. (**C**): Examples of RNAseq coverage plots for two exRTEs with distinct patterns of expression. (**D**): RT-PCR showing the expression of selected exRTEs in five-day-old seedlings.

We designed a workflow to identify LTR retrotransposons (RTEs) with high expression levels (Figure 1A). First, we predicted de novo 62,249 LTR-RTEs in the genome of the sunflower HA 412 HO line (v1.0, https://www.sunflowergenome.org/; [32]). Of these, we selected 35,013 RTEs (35 Kset) that were longer than 500 bp, and were similar to at least one of the canonical retrotransposon proteins (GAG, RT, RNAseH, AP, or INT).

Second, we used quality-filtered RNAseq reads from previous studies (Table 1) that sampled different tissues (leaves, roots, seeds, ovary, pistil, stamen, and ligule), under various osmotic stress conditions (NaCl treatment, 3 and 12 h; Polyethylene Glycol (PEG) treatment, 6 and 12 h) and hormone treatments (methyl jasmonate and abscisic acid (ABA) treatment). We mapped these reads to the genome, and allowed reads with as many as 200 hits to be reported. We selected 379 RTEs (1% of the 35 Kset) with reads per kilobase per million reads (RPKM) >0.25 in at least one sample. We manually checked the RNAseq read distribution along selected RTE sequences and found low coverage in most and high coverage in a few regions (mostly LTRs). This distribution of RNAseq reads may result from pervasive transcription (e.g., when only LTRs are covered) or transcription of other regions possessing partial RTE insertions (e.g., untranslated regions (UTRs) of genes) rather than expression of RTE *per se*.

To further decrease the number of such instances and identify true positive and highly expressed RTEs, we determined RTE sequence coverage by RNAseq reads and obtained 59 RTEs with coverages ≥60%. We manually curated the selected RTEs to filter out ones with short (<50 amino acids) RTE protein domain matches. We also removed highly similar (>95%) RTEs and obtained a set of 44 non-redundant and highly expressed RTEs (exRTEs). Of these, 19 exRTEs (42%) and 25 exRTEs were expressed in non-stressed and stressed conditions (Figure 1B). Some RTEs demonstrated tissue- and stress-specific expression patterns. For example, an exRTE (TE01s125448413HA412) of the Ty3/Gypsy superfamily was transcribed almost exclusively in non-stressed leaves and stamens and an exRTE (TE16s103520126HA412) of the Ty1/Copia superfamily was transcribed only in leaves under osmotic stress (Figure 1C). The results were verified by RT-PCR with primers designed on ten randomly selected RTEs and for nine (90%) of them amplification products were obtained (Figure 1D). Thus, we collected a set of tens of sunflower RTEs which are expressed in stress and non-stressed conditions.

### 2.2. Expressed RTE Set Is Enriched by “Young” Copies and Low-Copy Clades

**Figure 2:**
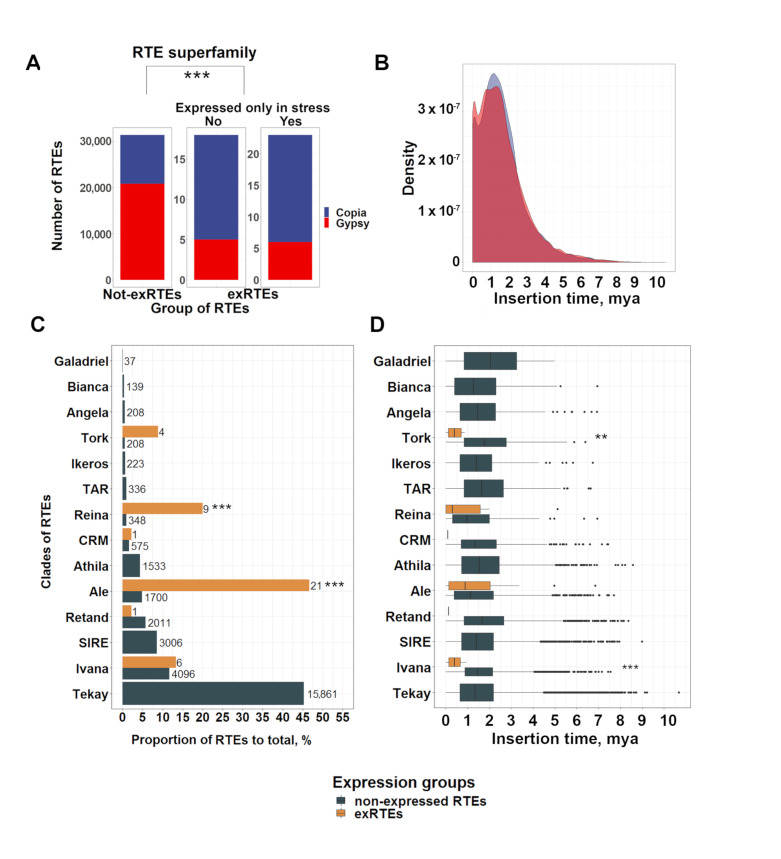
Classification and insertion time analysis in expressed LTR retrotransposons (exRTEs). (**A**): A bar plot comparing the number of Copia and Gypsy RTEs among exRTEs and non-expressed RTEs (n-exRTEs). Three stars indicate significant differences based on Fisher’s Exact Test for Count Data; *p*-value < 0.001. (**B**): Distribution of insertion time (mya: million years ago) values for *Copia* (blue) and *Gypsy* (red) superfamilies in the sunflower genome. (**C**): Clade-based classification of (n-)exRTEs. Three stars indicate significant differences based on Fisher’s Exact Test for Count Data; *p*-value < 0.001. (**D**): Insertion time (mya: million years ago) calculated for the individual (n-)exRTE clades. Two and three stars indicate significant differences based on Wilcoxon rank sum test *p*-value < 0.01 and < 0.001, respectively.

Based on protein domain homology to known RTEs, we classified 43 exRTEs into superfamilies and clades. Totally, of the 43 exRTEs, 70% belonged to the Ty1/Copia family, whereas only 34% of n-exRTEs were derived from this family (Fisher’s Exact Test for Count Data, *p*-value = 1.657 × 10^−7^, Figure 2A). We detected a significant enrichment of the Ty1/Copia superfamily in the exRTEs expressed in non-stressed conditions (Fisher’s Exact Test for Count Data, *p*-value = 0.0016) as well as exRTEs expressed only in stressed conditions (Fisher’s Exact Test for Count Data, *p*-value = 0.00017) when compared with non-expressed RTEs (n-exRTEs).

To establish whether these biases could be explained by the more recent activity common to Ty1/Copia members, we compared insertion times and identified no significant differences (Wilcoxon rank sum test with continuity correction, *p*-value = 0.6088; Figure 2B) between the two superfamilies.

We further classified RTEs and found that exRTEs belonged to six clades, including three from the Ty3/Gypsy (Reina, CRM, and Retand) and three from the Ty1/Copia (Ale, Ivana, and Tork) superfamilies. The exRTE set is significantly enriched (Fisher’s Exact Test for Count Data, *p*-value = 8.877 × 10^−13^) in Ale elements (Ty1/Copia superfamily), which account for almost half (21 exRTEs, 47.7%) of the exRTEs (Figure 2C). Reina elements (9, 20.5%) are also overrepresented in the exRTE set (Fisher’s Exact Test for Count Data, *p*-value = 2.925 × 10^−09^). Interestingly, members of the Tekay clade (Ty3/Gypsy), the largest (>15,000 copies) RTE family of the sunflower genome, were absent from the exRTE set.

We then tested the exRTEs of individual clades for biases in insertion time, compared with the n-exRTEs of the same clade. We found more recent insertion times for Ivana and Tork exRTEs (Figure 2D). Additionally, the insertion time distribution of all RTEs in the sunflower genome was bimodal: <0.5 Mya (recent insertions) and >0.5 Mya (late insertions). Twenty-one exRTEs (47.7%) belonged to the recently inserted RTE group, which is substantially higher (Fisher’s Exact Test for Count Data, *p*-value = 1.659 × 10^−7^) than the portion of n-exRTEs in this group (5932, 16.9%).

Together, these results showed that the exRTEs are recently inserted elements with significant biases toward the Ty1/Copia superfamily and low-copy RTE elements.

### 2.3. ExRTEs Domain Composition Is Biased Toward GAG Protein

**Figure 3:**
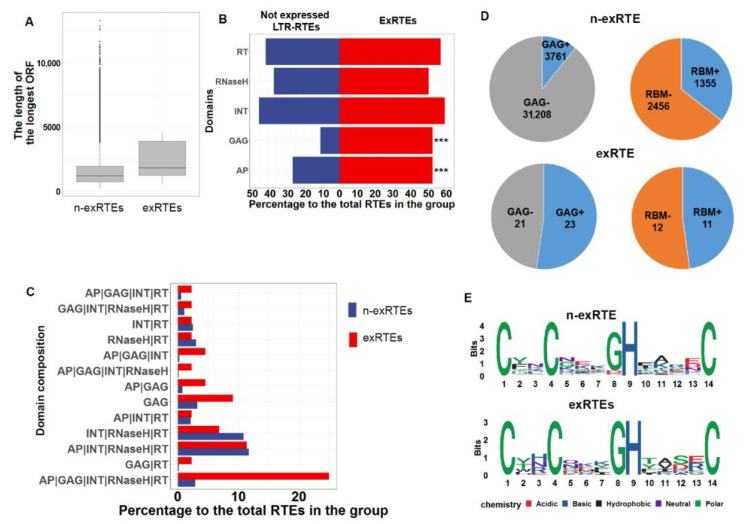
Open reading frame (ORF) length and domain composition analysis. (**A**): Boxplot comparing the distribution of maximum ORF length for exRTEs and n-exRTEs. A line in each box represents the median size of the longest ORF. Top and bottom edges of the box indicate the 75th and 25th percentiles, respectively. (**B**): Bar plot demonstrating the proportion of (n-)exRTE with predicted proteins exhibiting similarity to canonical RTE proteins. (**C**): Bar plot demonstrating the percentage of (n-)exRTEs with similarities to various combinations of canonical RTE proteins. (**D**): Pie charts demonstrating the number of (n-)exRTEs possessing ORFs that encode proteins with similarities to GAG (left), and the number of these proteins with and without RNA-binding motif (CX2CX4HX4C, right). (**E**): Logo of the amino acid sequence of the RBM motif of (n-)exRTEs GAG protein. *** indicates significant differences based on Fisher’s Exact Test for Count Data, *p*-value < 0.001.

We looked for differences between exRTEs and n-exRTEs in terms of the encoded proteins. We predicted all RTE ORFs and selected those with lengths >300 bp and similarities to one of five TE domains (GAG, INT, RT, RNAseH, or AP). We then compared the length and the number of ORFs for individual RTEs as well as the types of encoded proteins. We found no differences in the number of predicted ORFs (Wilcoxon rank sum test with continuity correction, *p*-value > 0.1). However, the exRTEs had longer ORFs (Wilcoxon rank sum test with continuity correction, *p*-value = 6.759 × 10^-5^; Figure 3A) and substantially differed from n-exRTEs with regard to the set of encoded proteins. Both groups contained a similar portion of RTEs that encoded RT, RNAse H, and INT proteins. However, a proportion of ORFs encoded GAG (23 exRTEs) and AP (23 exRTEs), which is significantly higher in exRTEs (Fisher’s Exact Test for Count Data, *p*-values were 1.138 × 10^-11^ and 0.0002487, for GAG and AP, respectively; Figure 3B) than in n-exRTEs.

We also compared the combination of RTE proteins encoded by selected ORFs for individual RTEs. Eleven (25%) exRTEs possessed one or more ORFs, together encoding all five proteins (Figure 3C) while the percentage of full-length RTEs among not-transcribed RTEs was significantly less (2.87%, Fisher’s Exact Test *p*-value = 3.682 × 10^-8^). In addition, other RTE protein combinations that contain GAG were overrepresented in exRTEs (Figure 3C), further highlighting GAG protein as a distinct feature of exRTEs.

To further explore GAG functionality, we screened all GAG-containing proteins for the presence of the RNA-binding GAG motif, CX2CX4HX4C, and compared the number of GAG proteins with and without this domain in exRTEs and n-exRTEs. We estimated a false-discovery rate (FDR) of 6% by dividing the number of ORFs which do not encode GAG, but which have the RNA-binding motif (RBM) (2670), by the total number of ORFs which do not encode GAG (45,393). We found that the proportion of the GAG-possessing RBM-encoding proteins was slightly higher in exRTEs than in n-exRTEs (47% (11 of 23) versus 35% (1359 of 3915); Figure 3D). We compared the number and sequences of RBM motifs in every GAG protein. All 11 of these exRTEs had a single RBM motif, whereas n-exRTEs had >1 RBM motif per GAG; the amino acid sequences of RBMs were similar between exRTEs and n-exRTEs (Figure 3E).

Thus, RTEs encoding GAG proteins with a single RBM motif are overrepresented among exRTEs, suggesting the importance of this protein for RTE RNA integrity.

### 2.4. The Proximity of exRTEs to Genes

**Figure 4:**
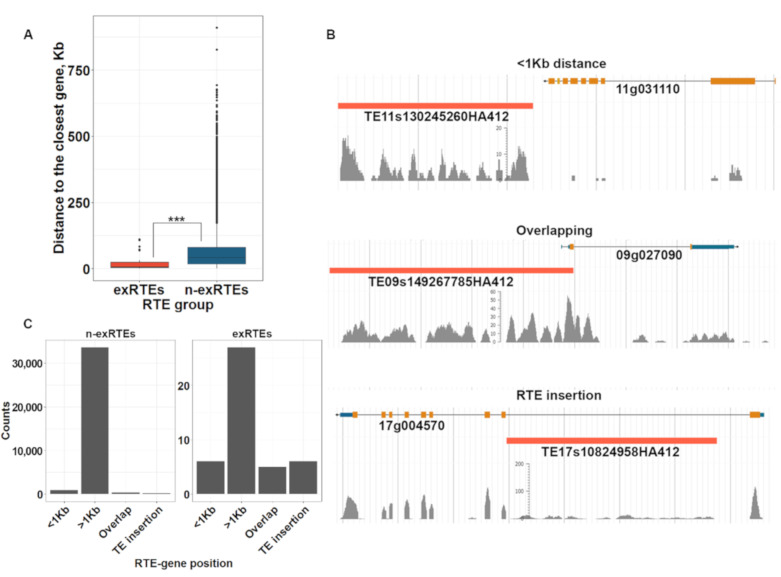
The distance of (n-)exRTEs to the adjacent genes. (**A**): Box plot of the distance (kb) to the closest genes for exRTEs and n-exRTEs. (**B**): Examples of exRTEs and their proximity to the closest genes. Three stars indicate significant differences based on Wilcoxon rank sum test *p*-value < 0.001 (**C**): Bar plot showing the number of (n-)exRTEs with different distances to the closest genes.

Proximity to neighboring genes is considered an important factor for RTE expression [12]. We analyzed the difference in proximity of exRTEs and n-exRTEs to the closest genes. ExRTEs tended to be significantly closer to annotated genes than n-exRTEs (Wilcoxon rank sum test, *p*-value = 1.065 × 10^−12^; Figure 4A).

We then classified all pairs of RTEs and their closest genes by the intervening distance: (1) distantly located (>1 kb distance between RTE and gene); (2) closely located (<1 kb distance between RTE and gene); (3) overlapped and (4) RTE insertion (RTE is found inside of annotated gene; Figure 4B). We found 27 exRTEs that were located >1000 bp from the closest gene and 17 exRTEs (38.6%) that were distributed among the other three categories (Figure 4C). Thus, the expression of only a few exRTEs were influenced by their being located close to a gene.

### 2.5. Most exRTEs Have Recent and/or Ongoing Mobilome Activity

To test the ability of exRTEs to generate new copies, we carried out a comparison of the relative copy number between sunflower cultivars. For this, we mapped previously obtained genomic Illumina reads of 13 sunflower cultivars [33] and estimated average coverage of exRTEs normalized by average coverage of five single-copy genes. For each retrotransposon, we obtained a ratio (ACM ratio) between the normalized RTE coverage and the minimum read coverage for this RTE across all cultivars. We assumed that exRTEs had additional copies in a cultivar if its ACM ratio was greater than 1.7, which is the maximum ACM value determined for the single-copy genes.

**Figure 5:**
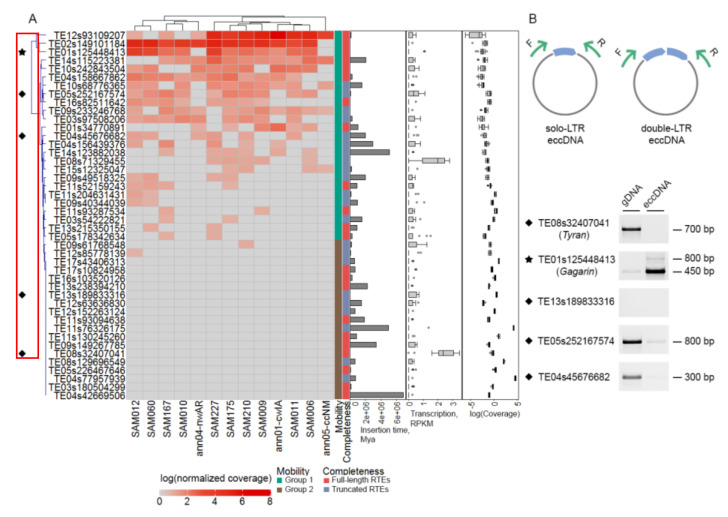
Mobilome activity of exRTEs. (**A**): Heatmap demonstrating the variation in ratio between the normalized RTE coverage and minimum read coverage for this RTE across 13 sunflower cultivars (ACM ratio, log2 transformed values are represented). Bar plot of insertion time values and boxplots showing distribution of reads per kilobase per million reads (RPKM) and normalized log_2_ transformed values of RTE coverage by genomic reads of 13 cultivars are drawn on the right side. (**B**): Inverted-PCR with total genomic DNA (gDNA) and genomic DNA enriched by extrachromosomal coiled DNA (eccDNA). Schema of primer annealing on eccDNA with single LTR (result of homologous recombination between two LTRs of RTE cDNA) and double LTRs (result of non-homologous end-joining of two LTRs of RTE cDNA) is represented in the top. Diamonds and stars indicate exRTEs used for eccDNA assay. Stars point to “*Gagarin*” RTE.

We observed that 25 (57%) exRTEs have additional copies in >1 cultivar, suggesting potential mobilome activity (group 1), while 19 (43%) exRTEs have additional copies in 0–1 cultivars (group 2; Figure 5A). It is worth noting that copy number estimation based on read coverage is strongly biased toward low-copy RTEs. To check for bias in copy number variation for exRTEs, we compared ACM values for group 1 and group 2 exRTEs. This analysis revealed a significantly higher ACM value for group 2 exRTEs, suggesting that our analysis may have lower sensitivity for high-copy RTEs. However, we cannot exclude the possibility that these results have biological rather than technical reasons and that they are caused by lower mobility of exRTEs with high copy numbers. No differences in transcription rates or insertion times were found between the two groups.

To detect ongoing transposition activity in exRTEs, we tested for the presence of extrachromosomal circular DNA (eccDNA) in five RTEs, including one with high and ubiquitous expression in all samples analyzed, TE08s32407041HA412. We isolated total genomic DNA from seedlings and specifically enriched it with eccDNA (see Materials and Methods). We conducted PCR with inverted primers (Figure 5B) and detected the amplicon with genomic DNA for four primer pairs; three of them showed significant depletion in eccDNA, suggesting that those primers annealed to genomic DNA rather than to eccDNA. Inverted PCR using primers that anneal to one exRTE—TE01s125448413, renamed “*Gagarin*”—demonstrated significant enrichment in eccDNA (Figure 5B). Moreover, we detected *Gagarin* eccDNA with single and double LTRs (Figure 5B). Notably, *Gagarin* is the first sunflower RTE with proven mobilome activity. *Gagarin* is expressed in leaves and stamens, demonstrating tissue-specific expression.

While our approach may underestimate the real number of exRTEs with the potential to create new copies, it clearly shows that more than half of the exRTEs have the potential to move and that at least one exRTE is capable of forming eccDNA in seedlings, implying ongoing transposition activity.

### 2.6. Nanopore Direct RNA Sequencing Revealed Alternative Splicing of exRTE Transcripts

**Figure 6:**
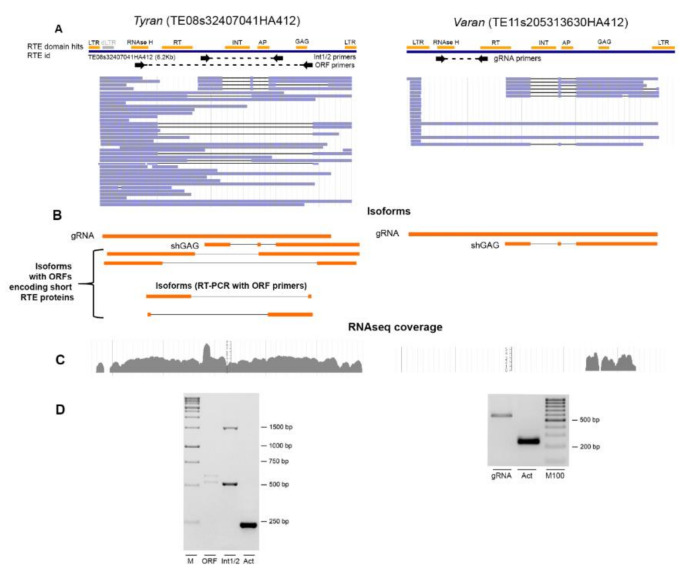
Detection of RTE transcripts by nanopore direct RNA sequencing (DRS). (**A**): Organization of the main protein domains and LTRs for Tyran (exRTEs, left) and Varan (RTE, right). DRS reads mapped to the genomic sequences of these RTEs are colored blue. Primer positions used for isoform verification are depicted below these. (**B**): Isoform composition for Varan and Tyran based on DRS read mapping and alignment of cDNA sequencing obtained with ORF primers (only for *Tyran*). (**C**): RNAseq (leaves) read coverage plot. (**D**): Results of RT-PCR with primers designed on *Varan* and *Tyran* isoforms and Actin (*Act*) in RNA extracted from 5-day-old seedlings.

Recent reports suggest that RTE life cycle may be dependent on RNA processing, including splicing [34]. To obtain the sequences of individual RTE transcripts, we performed nanopore Direct RNA (DRS) sequencing of poly-A+ enriched RNA of five-day-old seedlings. After base-calling, we obtained 380,000 reads. We mapped the reads to the sunflower HA412 genome sequence; the mapped positions intersected with predicted RTEs. We obtained evidence of expression of three RTEs including two exRTEs (TE01s34770891HA412 and TE08s32407041HA412) and one n-exRTE (TE11s205313630HA412). Of these, we identified the highest number of reads for two Ty1/Copia and full-length RTEs, TE08s32407041HA412, hereafter called *Tyran* and n-exRTE, TE11s205313630HA412, hereafter called *Varan*; Figure 6A,B). We verified their expression levels by RT-PCR (Figure 6D). The expression of Tyran was detected by our pipeline in all RNAseq experiments, but *Varan* was not, because of low coverage in RNAseq reads (Figure 6C). DRS reads provide a unique opportunity to study RTE isoform composition and splicing. We identified up to four *Tyran* and two *Varan* isoforms using DRS reads. Both RTEs encoded unspliced isoforms (gRNA); one isoform was short due to two spliced introns and the use of the alternative transcription termination site. The short isoforms of both RTEs encoded truncated GAG proteins which possess RBM of nucleocapsid domain but lack capsid domain as revealed by comparison of these proteins with EVD protein and HIV (human immunodeficiency virus) GAG protein. In addition, one *Tyran* isoform (“truncated”, Figure 6B) carries an ORF for RNAseH.

To discover additional isoforms, we performed RT-PCR with primers designed for the *Tyran* ORF. We obtained two bands of unexpected lengths (~540 and ~590 bp; Figure 6D). Sanger sequencing revealed that they correspond to the Tyran ORF with spliced internal sequences. These isoforms carried short ORFs (312 and 396 bp) that encoded truncated GAG and RT-RH proteins, respectively (Figure 6B). This indicated that our experiment underestimated the real number of isoforms produced by exRTEs. Thus, we used nanopore DRS sequencing and detected a set of full-length transcripts for sunflower RTEs and showed that they encode isoforms with different protein-coding potential.

## 3. Discussion

### 3.1. Tens of RTEs with Distinct Features Are Expressed in Plant Somatic Tissues

A growing body of evidence suggests that retrotransposons (RTE) are expressed in non-stressed somatic tissues. Although transposon-silencing mechanisms have largely been deciphered, it is unclear how some transposons are expressed when silencing system is on. Here, we applied an integrated approach to elucidate genomic and transcriptomic features as well as the mobilome activities of expressed RTEs of sunflower, a species for which retrotransposon expression has been demonstrated [26,27,28,29,30,31]. We observed 44 expressed LTR retrotransposons in the sunflower genome and studied their genomic and transcriptomic features and transposition activity. We determined that exRTEs are likely to have been inserted more recently than non-expressed RTEs (n-exRTEs). This may suggest that “genome immunity” against recently inserted RTEs is not well established, leading to their transcription. However, more than half ofthe exRTEs (23 exRTEs) were inserted >500,000 years ago. This finding is in line with recent observations of TE transcription across 12 vertebrate species, which showed that recent and ancient RTEs were both expressed in somatic and germline tissues [11]. Similarly, a global survey of RTE expression in maize did not reveal any biases in RTE insertion age among TEs producing poly-A transcripts [8]. Together, these results suggest that the link between RTE expression and insertion age is not straightforward and reasons other than temporal “blindness” of epigenetic-silencing systems against newly inserted RTE copies may exist.

We also observed that exRTEs were located close to genes. Previous findings in humans and plants showed the influence of retrotransposons on the transcription of neighboring genes [12,35,36]. The effect of this co-localization varied from complete silencing of the gene to co-option of the RTE sequence as a source of alternative transcription start or termination site. In turn, proximity to the gene may have a positive influence on RTE expression via different molecular mechanisms of expression activation. When an RTE is located close to the transcribed genes, its transcription can be a result of pervasive transcription or space extension of the regulatory mechanisms acting on the transcription of the neighboring gene. This may result in the coordinated expression of the RTE and its neighboring gene [12]. When the RTE is distantly located from the genes, the transcription may result from co-option of regulatory sequences such as stress response elements [30,37]. Overall, our results suggested that exRTEs are a diverse group of retrotransposons that lack unique patterns in terms of time of insertion or proximity to genes. Different mechanisms may regulate RTE expression and, more importantly, RTE transcript preservation in non-stressed somatic plant tissues.

### 3.2. The shGAG Isoform Originated via Splicing and Premature Transcription Termination Is Conserved Feature among Plant RTEs

We identified two GAG-related features for exRTEs: a more frequent occurrence of GAG ORFs and the generation of short isoforms (shGAG) carrying GAG ORFs by exRTEs. GAG binds RTE RNA via its RNA-binding motif (RBM), encapsulates the reverse transcription reaction, and protects RTE RNA from degradation by host defense systems such as siRNAs. To complete their life cycle, the number of GAG molecules must significantly exceed that of POL proteins [38]. Using nanopore DRS, we observed that two expressed RTEs, *Varan* and *Tyran*, produced short isoforms, carrying ORFs for GAG protein. These isoforms resulted from splicing and premature transcription termination. Similar shGAG isoforms have previously been observed in *Arabidopsis thaliana* EVD [34], barley BARE-1 [39], and *Drosophila melanogaster* [40] retrotransposons. However, proteins encoded by shGAG isoforms of Tyran and Varan are truncated and do not possess capsid domain of GAG. The capsid domain is essential for GAG oligomerization [41] suggesting that Tyran and Varan shGAGs are most probably not able to form virus-like particles and mobilize. It is also supported by our mobilome assay which showed no variation in copy number for Tyran between 13 sunflower cultivars. Moreover, no eccDNA was detected for Tyran. Importantly, shGAG proteins possess an RNA-binding motive (CX2CX4HX4C) as a part of nucleocapsid domain and may form a complex with RNA of the corresponding RTEs. We hypothesized that such an interaction may lead to sequestration of the corresponding RTE RNAs into RNA-protein particles protecting the RTE RNAs from degradation. From another side, truncated GAG protein can serve as dominant-negative factor limiting f RTE transposition activity. Such mechanism was previously described for yeast Ty1 retrotransposon [42].

We also found that whole exRTE set is significantly biased toward the presence of GAG ORFs and the typical GAG RNA-binding motif. GAG protein has low RNA specificity and may act in *trans* when binding to other RTE transcripts. Such a mechanism was shown for BARE-2 retrotransposon which does not encode its own GAG protein, but borrows it from its full-length counterpart, BARE-1 [39]. RTEs that only encode GAG are common in plant genomes [43]. We have provided evidence that such RTEs in sunflower provide an additional amount of GAG protein in the cell. The *trans* activity of GAG proteins and overrepresentation of GAG ORFs among expressed RTEs suggest that GAG may be a key player in providing flexibility and sustainability of the retrotranscriptome in a cell. However, further functional studies are required to confirm this hypothesis.

### 3.3. Ongoing Transcription and Transposition Activity Are Weakly Connected

More than 60% of our exRTEs exhibited copy number variation in at least one of 13 sunflower cultivars, suggesting recent transposition activity. Even in our small set, we were able to group exRTEs into three categories: (1) those with detectable expression and recent (copy number variation among 13 cultivars) as well as ongoing (detectable eccDNAs) transposition activity, including one exRTE called *Gagarin*; (2) those with detectable expression and recent transposition activity with no detectable ongoing activity; (3) those with detectable expression but without detectable transposition activity (e.g., *Tyran*). These results suggest that no direct links exist between retrotranscriptome and mobilome. This is in line with the analysis of RTE transcription in the *Arabidopsis* mutant *met1*, where the transcription of the EVD retrotransposon is triggered without observation of transposition activity [44].

Variability in RTE transcriptome-to-mobilome transition rate can be intraspecific. For example, analysis of ONSEN retrotransposon showed its expression in two *Vigna* cultivars while eccDNAs were detected in only one [45]. The transpositionally inactive copies may simply accumulate mutations which prevents cDNA formation. Alternatively, certain molecular mechanisms independent of post-transcriptional epigenetic silencing may regulate the retrotranscriptome to mobilome transition rate. The inhibition of RTE activity at the translation level was proposed a decade ago [44] and the role of UBP1 in this process was later demonstrated [46]. From an evolutionary point of view, the existence of such mechanisms can be beneficial for host and allows the co-option of RTE transcripts serving as a reservoir for evolutionary novelties. Indeed, a growing body of evidence suggests that the RTE(-derived) transcripts play important roles in regulation of gene expression. For example, a number of protein-coding transcripts and long non-coding RNAs have RTE-derived sequences and RTE transcripts can modulate their expression (reviewed by [47]). The evolutionary advantages that some RTE transcripts confer on the host result in relaxation of silencing and rise retrotranscriptome-to-mobilome transition rate. It will be interesting in the future to check whether retrotranscriptome-to-mobilome transition rate for transcriptionally active plant RTEs (e.g., *Tyran* and *Varan*) but with no detectable transposition activity is raised in a stress- or developmental stage-dependent manner.

### 3.4. RNAseq and Nanopore RNA Sequencing Are Complementary Approaches for Identifying Expressed RTEs

The vast majority of previous studies of RTE expression used short RNAseq reads to identify expressed RTEs. Although RNAseq is a robust method of gene expression analysis, it is challenging to estimate retrotransposon expression using this approach [8,25,48]. Our first attempt to detect expressed RTEs by RNAseq led to the identification of >300 expressed RTEs. However, manual curation of the read-coverage profile revealed that most RTEs have few regions with significant coverage in RNAseq. Such patterns may result from pervasive transcription from neighbor sequences (e.g., gene promoters) or if the RNAseq reads originate from other transcribed sequences (e.g., UTR of the genes) where the RTE regions persisted. To overcome these challenges, we applied an RTE coverage cutoff, which reduced the number of expressed RTEs nine-fold and allowed us to detect 44 expressed RTEs in sunflower with RT-PCR verification.

Our coverage-based approach has several limitations. First, we anticipate that it detects only highly expressed RTEs and filters out those with low expression. Thus, we may have underestimated the number of expressed RTEs in this study. Second, RTEs located in alternative introns will not be filtered out. We decided to retain such instances (seven exRTEs) because we were uncertain if the RNAseq coverage of intronic exRTEs was due to their independent expression or due to intron retention. Third, some exRTEs have more than one near-identical copies in the genome and the RNAseq reads covered all of them almost equally. If the copies are sufficiently divergent, the information about primary and secondary read alignments can be used to determine which copy is expressed. For example, *Tyran* has two copies, on chromosome 8 and 14, but most (98%) of the primary alignments were assigned to chromosome 8. Fourth, the biggest drawback of an RNAseq-based study of RTE transcription is its inability to reconstruct individual RTE transcript sequences. To sequence RTE transcripts, we used nanopore DRS, which allowed sequencing of single RNA molecules. Using DRS reads, we unambiguously identified full-length reads for *Tyran*, the most highly expressed RTE in our dataset. Surprisingly, we identified one predicted RTE called *Varan* whose RNAseq-estimated expression was not sufficient to include it in the exRTE set.

One of the limitations of nanopore DRS is low yield of reads. We were able to generate 380,000 sunflower RNA reads that is lower than previous reports on Arabidopsis (~1 million reads per library [49]) and moss (*Physcomitrium patens*, 1.3–1.8 million reads per library [50]) but comparable with the results on humans (50,000–831,000 per flow cell [51]). The number of obtained nanopore reads was substantially lower than the number of Illumina reads used in our study (>13,000,000 reads per sample). However, comparable number of Illumina and nanopore RNAseq reads is required for detection of similar number of transcripts [52]. Therefore, we suggest that low number of obtained DRS reads is one of the reasons why most of the exRTEs identified by our Illumina RNAseq based pipeline (Figure 1A) was not detected by nanopore DRS. Thus, RNAseq and nanopore RNA- or cDNA-sequencing should be considered complementary approaches for detecting ex-RTEs, where the former provides deep transcriptome coverage, and the latter provides superior transcript-level resolution.

## 4. Materials and Methods

### 4.1. Plant Material and DNA Isolation

Seeds of sunflower cultivar “Enisey” (“Gavrish” seed company, Moscow, Russia) were germinated in dark (room temperature) over 5–7 days. Genomic DNA was isolated from the seedlings following the CTAB (cetyltrimethylammonium bromide) protocol [53].

### 4.2. Pipeline for Genome-Wide Detection of Highly Expressed TEs

LTR retrotransposons (RTEs) were predicted in the sunflower genome HA 412 HO line (v1.1, https://www.sunflowergenome.org/) using LTRharvest 1.5.10 with default parameters [54] followed by RTE domain identification by LTRdigest 1.5.10 [55] with the following parameters: -aaout yes-pptlen 10 30 -pbsoffset 0 3 -pdomevalcutoff 0.001. Hidden Markov model based (HMM) profiles of RTE domains for LTRdigest were retrieved from the GyDB database [56]. The gff3 file from LTRdigest analysis was parsed using custom python script (https://github.com/Kirovez/LTR-RTE-analysis/blob/master/LtrDiParser_v2.2.py) to extract sequences of LTR retrotransposons possessing similarity to any RTE domains including GAG, reverse transcriptase, RNAse H, aspartic protease and integrase. The sequences of RTEs were then used for classification using TEsorter software (https://github.com/zhangrengang/TEsorter) with default parameters. Publicly available RNA-seq data were retrieved from NCBI SRA (Table 1). Adaptor trimming and read quality filtering were performed with trimmomatic v0.38 [57]. For each run, reads were aligned to the sunflower genome with HISAT2 [58] using option “-k 200” to allow multihit mapping. The obtained sam file was converted to bam file by samtools [59] (samtools view -Sb) followed by sorting of bam file by bamtools [60]. TE expression (fragments per kilobase of transcript, per million fragments sequenced (FPKM)) was estimated by StringTie2 [61]. Low expressed TEs with (<0.25 FPKM) were filtered out. To calculate TE coverage by RNAseq reads the sorted bam files were intersected with RTE coordinates using the coverageBed tool [62] with the parameters: -split -histintersect. The obtained hist file was parsed by custom script (https://github.com/Kirovez/LTR-RTE-analysis/blob/master/SelectTEbyCoverageFromHist.py) with parameters: 1 0.6 to find RTEs with a >0.6 coverage by RNAseq reads. All data on identified RTEs are included into Supplementary Table S1.

### 4.3. RTE Insertion Time Estimation

For the insertion time analysis, a python script was written (https://github.com/Kirovez/LTR-RTE-analysis/blob/master/TEinsertionEstimator.py). For all RTEs, the insertion time was calculated by the following formula T  =  k/2r, where k is the distance between LTRs estimated using Kimura’s two-parameter (K2P) model [63] and r is the mutation rate. The mutation rate 1.3  ×  10^−8^ substitutions per site per year [64] was used. Parameter K was calculated as 0.5 * log((1–2p -q) * sqrt(1–2q)), where p is transition frequency and q is transversion frequency. The transition and transversion frequencies were estimated after the alignment of 5′ and 3′ LTR sequences by clustalw2 software (http://www.clustal.org/clustal2).

### 4.4. Search of RNA-Binding Motif and GAG ORF Analysis

For identification of the RNA-binding motif (CX2CX4HX4C, where X is any amino acid) in GAG proteins, we predicted all ORFs and their corresponding proteins for identified RTEs using getorf program of the EMBOSS suite [65] with parameters “-minsize 300 -find 1”. The obtained fasta file was blasted against the RExDB database [66] to obtain a list of GAG protein ORFs that were also used for the ORF length comparison. Sequences of all predicted proteins were used for identification of RBM motif using custom python script (https://github.com/Kirovez/LTR-RTE-analysis/blob/master/RBM_GAG_screen.py). Proteins with no similarity to GAG were used to calculate FDR.

### 4.5. Calculation of RTE—Gene Distance

To calculate distance between the closest genes and RTEs, gff3 genome annotation file was downloaded from https://www.heliagene.org/HA412.v1.1.bronze.20141015/. The coordinates of RTEs in the genome and the gff3 genome annotation file were used for calculation by custom script (https://github.com/Kirovez/LTR-RTE-analysis/blob/master/RTE_gene_distance.py).

### 4.6. Mobilome Analysis

To estimate copy number variation of exRTEs, publicly available genomic data for 13 sunflower cultivars [33] were downloaded from NCBI (Table 2).

The RepeatProfiler (https://github.com/johnssproul/RepeatProfiler) tool was used to calculate normalized coverage of 44 exRTEs by genomic reads. For normalization, five single-copy sunflower genes (Supplementary Table S1) were used. These genes were identified by blastn search of all sunflower genes versus the genome followed by selection of the genes with a single hit. For each RTE and reference gene, we obtained a ratio between the normalized coverage and the minimum read coverage across all cultivars (ACM ratio). To determine ACM cutoff above which an RTE is accounted as having transposition activity, we determined maximum and minimum ACM values for each reference gene across data from 13 cultivars. Then, for each reference gene the maximum ACM value was divided by the minimum ACM value resulted in the ACM cutoff for RTEs (~1.7).

### 4.7. RNA Isolation and RT-PCR

RNA was isolated from 5-day-old seedlings of sunflower cultivar “Enisey” (“Gavrish” seed company, Moscow, Russia) using an ExtractRNA kit (Evrogen, Moscow, Russia) following the manufacturer’s instructions. The RNA concentration and integrity were estimated by Nanodrop (Nanodrop Technologies) and gel electrophoresis using an 1.2% agarose gel with ethidium bromide staining. The RT-PCR was carried out using a BioMaster qRT-PCR (2×) reagent kit (Biolabmix, Novosibirsk, Russia) and primers (Table 3). Primers flanking an intron of the actin gene were used as reference and as additional controls of DNA contamination. PCR reactions on MQ and DNAse-treated RNA was carried out as negative controls. The PCR products were visualized by gel electrophoresis on an 1.2% agarose gel with ethidium bromide staining.

### 4.8. Extrachromosomal Circular DNA Isolation

The procedure of eccDNA enrichment was performed according to Lanciano et al. [16] with several modifications. Briefly, eccDNA was isolated from 5 µg of genomic DNA using Plasmid-Safe ATP-Dependent DNAse (Epicenter, Madison, WI, USA) according to the manufacturer’s instructions. Treatment duration was extended to 48 h. DNA precipitation was performed by adding 0.1 volume 3 M sodium acetate and 2.5 volume absolute ethanol followed by overnight incubation at −20 °C. After centrifugation, eccDNA pellet was obtained and exposed to RCA (rolling circle amplification) reaction by Illustra TempliPhi 100 Amplification Kit (GE Healthcare) for 65 h at 28 °C. Detection of TE eccDNA was performed by inverse PCR with specific primers (Table 4).

### 4.9. Nanopore Direct RNA Sequencing and ONT Data Analysis

Purification of mRNA was conducted from 75 ug of total RNA using Dynabeads mRNA DIRECT Kit (ThermoFisher Scientific, Waltham, MA, USA) following the manufacturer’s instructions. To assess the concentration and quality of mRNA, a Quantus Fluorometer (Promega Corporation, Madison, WI, USA) and gel electrophoresis were used. The library was prepared from 1 ug poly(A)+ using the Nanopore Direct RNA Sequencing Kit SQK-RNA002 (Oxford Nanopore Technologies, Oxford, UK). Sequencing was performed by MinION using flow cell FLO-MIN106. Reads were basecalled using Guppy (Version 4.0.11). Reads were mapped to the sunflower genome by minimap2 software [67] with the “-ax splice” argument. The obtained sam file was converted to a bam file by samtools [59] (samtools view -Sb) followed by sorting of the bam file by bamtools [60]. Transcript assembly was performed by StringTie2 [61] with the following arguments: -L -j 2 -f 0.05. The obtained gtf file was converted to gff by gffread tool [68]. The sorted bam and gff files were used for read mapping visualization by locally installed JBrowse [69].

### 4.10. Statistics and Data Visualization

Statistical analysis was carried out in Rstudio Version 1.2.1335 (http://www.rstudio.com/) with R version 3.6.0. Visualization was carried out by ggplot2 [70] (bar plots, density plot and boxplots), ComplexHeatmap [71], and VennDiagram [72] packages. Logo of RBM motives was drawn by ggseqlogo [73].

## Figures and Tables

**Table 1:** NCBI accession numbers of RNAseq read archives used in this study.

Accession Number in NCBI	Description	Number of Reads after Quality Filtering
SRR7691052	Pistil	19,498,261
SRR7691053	Stamen	21,945,535
SRR7691054	Ligule	21,602,172
SRR7691055	Leaf	20,908,077
SRR7691059	NaCl 12 h	23,081,013
SRR7691051	NaCl 3 h	21,727,606
SRR7691057	Seeds	22,697,764
SRR7691056	Roots	18,190,537
SRR7691047	PEG 12 h	20,937,047
SRR7691048	PEG 6 h	23,482,045
SRR4996808	Ovary	13,636,857
SRR4996851	ABA leaves	31,312,818
SRR4996849	Meja leaves	20,970,863

**Table 2:** Sequence Read Archive (SRA) accessions of genomic reads of 13 sunflower cultivars [33].

SRA	Cultivar
SRR10484607	SAM227
SRR10484608	SAM060
SRR10484609	SAM167
SRR10484610	SAM175
SRR10737894	SAM210
SRR5140325	SAM012
SRR5140331	SAM011
SRR5140336	SAM010
SRR5140395	SAM006
SRR5907847	ann04-nwAR
SRR5907848	ann05-ccNM
SRR5907869	ann01-cwIA
SRR5912489	SAM009

**Table 3:** RT-PCR primers.

Gene/TE ids	Primers
Actin	TTCAACGTTCCCGCCATGTA; GTTCGGCAGTGGTTGTGAAC
TE01s125448413	ATTGGCTTCGATCCATCTCGACG; AGATGTAGGGAAACGGGTGGAGT
TE04s156439376	CACTGTGACTTGTGGACATCCCC; GACGAATCATGCGCTCGGATTTC
TE05s178342634	CCGGGTCAACCTGTCATGGATTT; TGGGCATCCTAAATTGTGTGGCA
TE13s189833316	ACCACTTAGCAGCACAAACTCGT; GGTAACCGACATGCCTTCCTTCA
TE05s252167574	AGCCGTACAGAGACGAAGAGACA; TTTGCCCACCAGGTTGATGCATA
TE09s40344039	GATCTGGAGCATGCGTATGGAGG; GTGGCCGCCTTAGAAGCAATAGA
TE04s45676682	TACCAGCAAGAATTTGAGCGGCT; GGCGGTCACGTATTTCTGCACTA;
TE03s54222821	TAGAACTCTTGCTAGGGCGTGGA; TCTGGGAAGATTTGGTGCAAGCA
TE08s71329455	GATGGGTGATGGTTCGGGTGAAA; CGGACCAAACTTCTGCTGCCTAT
Int1/2 (Tyran)	CCAGTCACCAGGATTCTCCC; GATTCGAAATCAGGGAGAATC
ORF (Tyran)	AGGGTGATAGTTCTGGGTCCT; GGAACACAGGGTTAGCTGCT
gRNA (Varan)	CTGTTTCAGCCCATACAGCGACT; GGTCCTCTAGAACTTCTGTTGCTCC

**Table 4:** Primers for eccDNA amplification.

Gene/TE Id	Primers
Tyran	TCACTTGCTTGGAGATATGGGT; TCCTCACTACCCCGACTTCA
Gagarin	CGAAGAGGCTACTTGGGAGA; CGGACTGGATTTCTTGCATT
TE13s189833316	CAAAACCCGCTTCAAAGAAA; CAGCCCCTTGTGTCTTCCTA
TE05s252167574	GGTGAGGTTGACGGTGGTAT; GGAGTCGAAACGGAATGTGT
TE04s45676682	GGATTTGTTTGTTTTAATGTGATG; TGGAATTCAGCATTGGTACG

## Data Availability

Nanopore data produced for this study is available in SRA (NCBI) under Bioproject Accession PRJNA681689.

## Supplementary Materials

The following are available online at https://www.mdpi.com/1422-0067/21/23/9331/s1. Table S1: General information about identified sunflower retrotransposons; File S1: Fasta sequences of single-copy sunflower genes used for mobilome assay.
